# Editorial: Human health affected by changing ecological environment in the rapid urbanization

**DOI:** 10.3389/fpubh.2026.1782377

**Published:** 2026-03-12

**Authors:** Yunhui Zhang, Md Galal Uddin, Jing Yang, Domenico Cicchella

**Affiliations:** 1 Faculty of Geosciences and Engineering, Southwest Jiaotong University, Chengdu, Sichuan, China; 2 School of Engineering, University of Galway, Galway, Ireland; 3 Department of Neurology, West China Hospital of Sichuan University, Chengdu, China; 4 Department of Science and Technology, University of Sannio, Benevento, Italy

**Keywords:** development trend and optimization, environmental contamination, human health, influencing factors, urbanization

The migration of people from rural to urban areas is driving profound social transformations worldwide. Today, cities are home to more than half of the world's population and generate over two-thirds of global GDP, with projections indicating that by 2050, approximately two-thirds of the global population will live in urban areas. While fostering economic growth, rapid urbanization is also creating substantial ecological pressures (pollution, increased waste, soil degradation, water scarcity, and worsening air quality), threatening public health and ecosystem balance. The health impact of this process has often been compared to the transition to agriculture during the Neolithic period, which, while driving population growth through food surpluses, was associated with declines in several health indicators (1). Evidence suggests increases in malnutrition, reductions in average height and decreases in life expectancy (2). Epidemics increased significantly: zoonoses, intestinal parasites, and respiratory infections spread rapidly, increasing infant mortality (3).

Today, to avoid these problems and better address the public health consequences of rapid urbanization, scientific research must serve as a valuable reference point for policymakers, guiding them in the development and adoption of resilient policies.

To this end, a multidisciplinary scientific approach is essential. Monitoring exposures and integrating environmental and clinical data are critical steps, as understanding these interconnections is not only a scientific priority but also an ethical imperative.

This Research Topic brings together 14 contributions (11 original research articles, two reviews, and one opinion article) that explore, from complementary perspectives, the relationship between environment, urbanization, and health, together outlining a complex framework where pollution, climate, urban planning, and public policies interact to determine health outcomes.

A first group of articles examines the direct effects of environmental pollutants on health. Du et al. provide a review of the role of environmental particulate matter (tobacco smoke, coal combustion particles, and heavy metals) in the development of dental caries. The study's main strength is its in-depth analysis of biological mechanisms, including oxidative stress, apoptosis, and alterations in the oral microenvironment.

Still in relation to air pollutants, He et al. report that exposure to even low levels of carbon monoxide (CO) is associated with short-term adverse effects related to pulmonary tuberculosis in developing regions. This finding is particularly alarming because it suggests the absence of a clear safety threshold. However, the observational nature of the study limits the ability to fully exclude socioeconomic confounding factors.

Yan et al. and Li et al. focus on heavy metals. Yan et al. highlight that arsenic in rice from Henan Province represents the main health risk, with greater vulnerability for children and infants; they also report higher cadmium levels in urban areas. Li et al. link metal exposure, measured by urinary concentrations, to the risk of non-alcoholic fatty liver disease (NAFLD), suggesting that inflammation and metabolic dysfunction mediate this association. The utility of these studies lies in integrating biomonitoring and clinical assessment, although longitudinal confirmation is needed to establish causality.

Liu et al. extend this perspective by investigating the role of phthalates in overactive bladder and finding a positive correlation between urinary concentrations and symptoms. This is a preliminary yet meaningful finding that generates new pathogenetic hypotheses; the main limitation is the need for larger cohorts and prospective designs.

A second group of studies links climate conditions and infectious diseases. Hyrkäs-Palmu et al., in a systematic review, show that low temperatures and high population density increase the incidence of respiratory infections in urban areas, highlighting the interaction between meteorological factors and the built environment. Su et al. highlight that extremely high temperatures are associated with an increase in hospitalizations for infectious pneumonia.

In parallel, Wang W. et al. observe greater viral abundance in urban centers compared to rural areas and identify SO_2_ and wind speed as determining factors in the distribution of viruses.

These studies are particularly useful in the context of climate change, as they show how both extreme cold and heat can increase vulnerability to infections. However, the complexity of ecological and social systems makes it difficult to isolate the specific effect of each environmental factor.

Li and Xu analyze health risks related to high temperatures in eastern China, showing a concentration of risks in central urban areas and an overall improvement from 2010 to 2025. The study provides an important contribution to spatial epidemiology, but the distribution models, while sophisticated, remain dependent on the quality of available environmental and health data.

Amin and Bertelsen, in an opinion article, broaden the perspective to indoor environments, describing how climate change modifies microbial exposure through interconnected pathways: meteorological changes, transformations of the environmental microbiome, architectural adaptations, and allergen dynamics. While it does not provide original empirical data, the article has the merit of integrating different disciplines and drawing attention to often-overlooked emerging risks.

A third group of articles concerns the impact of urbanization and land-use policies. Peng et al. demonstrate that AI-based smart city policies improve both green economic efficiency and public health outcomes, with effects varying depending on regions' technological innovation capacity.

Wang Z. et al. emphasize the need, during rapid urbanization, to strengthen health services, rationally plan land use, and protect vulnerable populations. Wu et al., using data from the China Family Panel Studies, show that *in-situ* urbanization has positive effects on individual health, mediated by increased household income. Finally, Yang et al. analyze the relationship between urban-rural integration and air quality in the middle reaches of the Yangtze River, highlighting progress but also significant regional imbalances.

These studies have high practical utility: they provide empirical foundations for policy decisions and health-oriented urban planning. However, many findings are mediated by socioeconomic variables and are context-specific to the Chinese context, which may limit their generalizability beyond it. Further studies in different geographical contexts are needed to validate and broaden the generalizability of the findings, strengthening their global relevance. Overall, these articles demonstrate how health and the environment are closely interconnected, along a continuum that extends from molecular exposure to pollutants to urban macro-policies. Their main strength is their multidisciplinary and multilevel approach; their common limitation is the difficulty of establishing definitive causal links in complex and rapidly evolving systems. Nonetheless, they provide crucial insights to guide prevention, planning, and public policies in an era marked by accelerated urbanization and climate change. To outline more ambitious future research avenues, it would be useful to develop investigations that integrate rigorous causal inference methods, longitudinal cohorts of heterogeneous, geographically diverse populations, and the innovative use of environmental and genomic big data. These approaches will not only overcome the limitations of current cross-sectional studies but also clarify the causal dynamics between environmental contamination and health outcomes, providing a solid foundation for effective mitigation policies globally.
